# High copper levels induce oxidative stress and inflammatory processes in a cell culture model of Wilson’s disease

**DOI:** 10.1007/s11010-026-05481-6

**Published:** 2026-01-22

**Authors:** Martha-Julia Sasula, Anna T. J. Held, Stefan Schefczyk, Marcin Krawczyk, Andree Zibert, Hartmut H. Schmidt, Ruth Broering

**Affiliations:** 1 Department of Gastroenterology, Hepatology and Transplant Medicine, Medical Faculty, University of Duisburg-Essen, Hufelandstr. 55, 45147, Essen, Germany; 2 Department of Internal Medicine B, University Hospital Muenster, Muenster, Germany

**Keywords:** Wilson disease, ATP7B mutation, NFKB, AP1, ROS, Inflammation

## Abstract

Wilson’s disease (WD) is a genetic disorder resulting from mutations in the *ATP7B* gene that lead to copper overload in hepatocytes. To gain a deeper understanding of the cellular mechanisms underlying WD, a reanalysis of the GSE1073236 dataset was conducted and validated by comparative analyses in a cell culture model of HepG2 and HepG2 ATP7B knockout (ATP7B-KO) cells, as well as in primary hepatocytes from WD patients and controls. Increased expression levels of genes associated with autophagy, oxidative stress and inflammation were observed in copper-treated HepG2 ATP7B-KO cells and in primary hepatocytes from patients with WD. Furthermore, copper increased the secretion of IL1B, TNF, GM-CSF, and IL8 in HepG2 ATP7B-KO cells. Accordingly, copper exposure enhanced NFKB promoter activity 6 h after treatment. However, the transcriptional activity of AP1 and NFKB was reduced in these cells 24 h after treatment with 0.6mM CuCl_2_. In addition, both HepG2 and HepG2 ATP7B-KO cells showed increased oxidative stress and H_2_O_2_ levels after copper treatment, indicating that reactive oxygen species could play a role in WD. In the WD cell culture model, critical mechanisms behind copper-related cell death highlight the importance of this model in developing molecular targets for future therapeutic strategies.

**Graphical abstract:**

**Figure:**
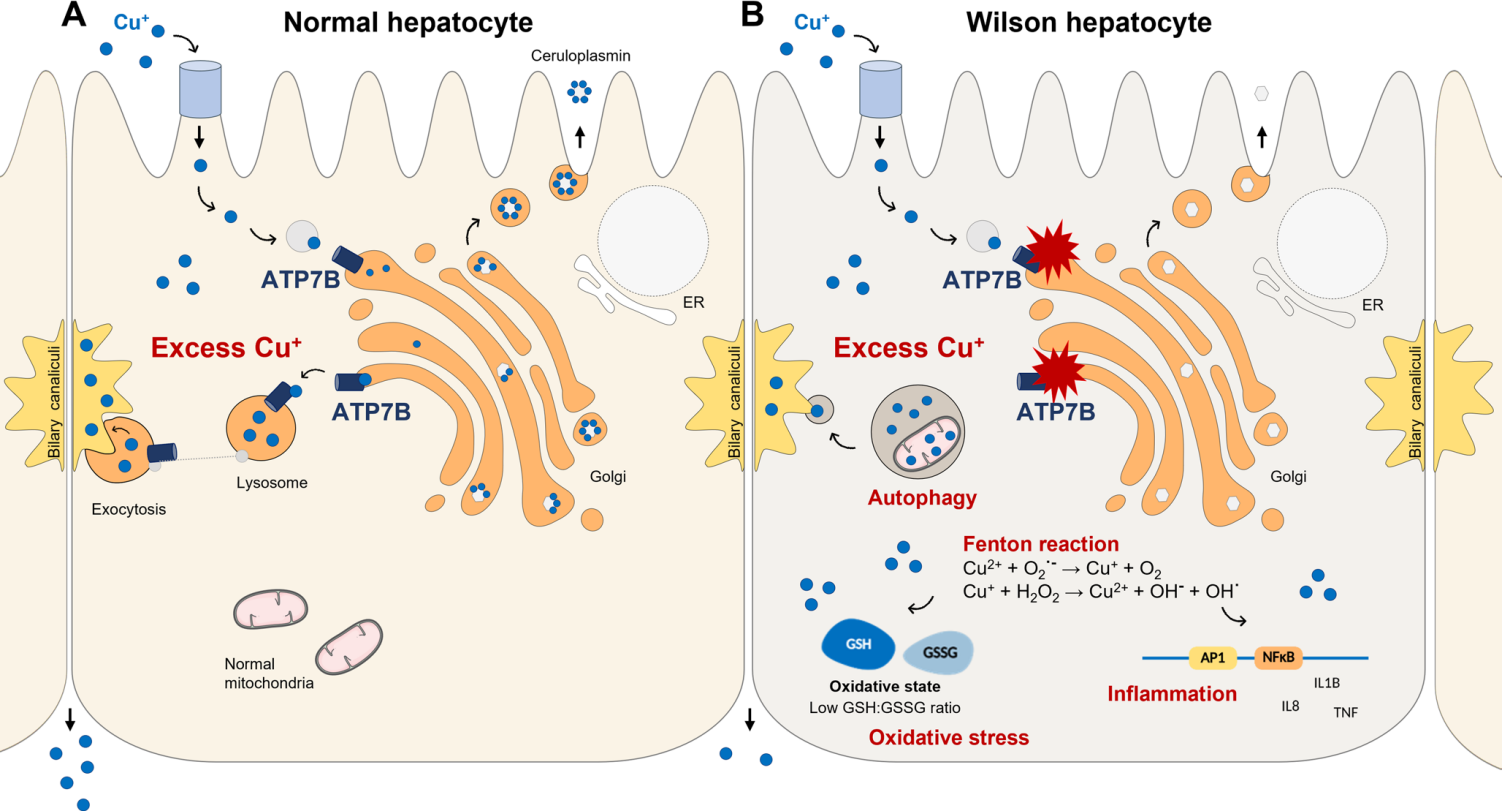
**A** Copper is transported through APT7B in the Golgi apparatus where it is loaded onto ceruloplasmin for secretion. In case of excess copper, it is released into bilary canaliculi by ATP7B via lysosomes and exocytosis. **B** In Wilson disease various mutations in the ATP7B gene lead to a loss of function of the protein causing a copper build-up that triggers autophagy, oxidative stress, and inflammation, driven in part by altered NFKB and AP1 signalling. This study highlights how copper overload promotes cellular damage and inflammatory responses, deepening the understanding of the molecular mechanisms underlying Wilson's disease

## Introduction

Wilson’s disease (WD), which leads to excessive accumulation of copper, is an autosomal recessive disorder with a potentially life-threatening outcome and an incidence rate of 1:30,000 [1]. The *ATP7B* gene encodes an intracellular copper transporter that is highly expressed in hepatocytes. WD-associated dysfunction of ATP7B results in cellular copper accumulation, which primarily affects liver and brain function. Excessive copper levels in the liver have been demonstrated to result in a number of physiological consequences, including mitochondrial dysfunction, the downregulation of copper influx, the upregulation of metallothioneins (MTs), and ultimately, cell death. Consequently, hepatocytes attempt to repair cellular damage through increased autophagy and mitophagy, which ultimately fails [2]. Copper transporter deficiency in ATP7A-deficient Menkes disease-mimicking fibroblasts is associated with mitochondrial redox imbalance that contribute to toxicity, related to copper overload [3]. A comprehensive understanding of the complex pathophysiology in WD cells may result in the identification of potential direct-acting therapeutic targets.

Copper is an essential trace element that plays a crucial role in numerous biological processes. As a constituent element of enzymes and proteins, copper regulates diverse biochemical processes, including energy production, the regulation of transcription factors, and the induction of reactive oxygen species (ROS) [3, 4]. The identification of the mechanisms that contribute to or mitigate copper toxicity in WD is highly important. Suppression of copper transporter ATP7B has been linked to alterations in intracellular copper trafficking as well as lipid metabolism and cell cycle machinery in an Atp7b-/- mouse model [5, 6]. To address copper-dependent toxicity in WD in human liver cells, we reanalysed the RNA sequencing data (GSE107323) [2], validated gene expression signatures and functionally addressed our findings in the HepG2 ATP7B knockout (ATP7B-KO) model [7]. This study aims to address known WD-associated cellular mechanisms in this cell culture model, increasing its significance for the development and testing of direct-acting therapeutic targets.

## Materials and methods

### Primary human hepatocyte isolation

Primary human hepatocytes (PHH) were prepared from nontumorous tissues obtained from freshly resected human livers of different donors (PHH Ctrl, *n* = 4) or explanted livers of WD patients (PHH WD, *n* = 2) via a two-step perfusion protocol as previously described [8]. All patients gave a written declaration to which they had provided consent. The study was conducted in accordance with the ethical guidelines set out in the 1975 Declaration of Helsinki and received approval from the institutional review board (ethics committee). Human biological samples and associated data were provided by the Westdeutsche Biobank Essen (WBE, University Hospital Essen, University of Duisburg-Essen, Essen, Germany; approval 18-WBE-048).

### Cell culture

HepG2 cells (ATTC-HB-8065, lot no. 70010583) and HepG2 ATP7B-KO cells [7] were cultured in RPMI medium supplemented with 10% fetal bovine serum, 100U/ml penicillin, 10 µg/ml streptomycin and 2mM L-glutamine. Cells were cultured under sterile conditions at 37 °C, 5% CO₂ and 95% humidity and used for experiments at 70–80% confluence. HepG2 ATP7B-KO cells have been generated by nucleofection of a custom-made CompoZr^®^ zinc finger nuclease plasmid (Sigma; #CKOZFND3740-1KT) into HepG2 cells [7].

### Cell viability assay

Cell viability of CuCl_2_ (Merck) or CuSO_4_ (Merck) -treated HepG2 and HepG2 ATP7B-KO cells was measurement by cell counting assay (CCK-8, Sigma-Aldrich). Calibration curves for HepG2 and HepG2 ATP7B-KO cells were generated according to manufacturer’s instructions by plating 40,000, 20,000, 10,000, 5,000, 2,500, 1,250, 652, 312, 156 and 0 cells in 100 µl Medium without CuCl_2_ and CuSO_4_ in a 96-well-plate. After 24 and 48 h medium was changed. For toxicity measurement 20,000 cells were plated in duplicates in 100 µl medium for 24 h. Medium was changed containing different concentrations of CuCl_2_ or CuSO_4_ (0, 0.2, 0.4, 0.6, 0.8, 1.0, 1.2mmol/l). After 24 h medium was discarded as recommended in [9] and 110 µl medium without CuCl_2_ or CuSO_4_ containing 10 µl CCK-8 Solution was added. Cells were incubated for 2 h at 37 °C. Absorbance was measured at 450 nm by a microplate reader (Multi-mode microplate reader FLUOstar Omega, BMG LabTech GmbH). Cell viability was calculated using the calibration curve. Untreated cells were used as control (100%).

### RNA isolation, cDNA synthesis and quantitative PCR in HepG2 cells

For RNA isolation 50,000 cells (HepG2 and HepG2 ATP7B-KO) were plated in 800 µl Medium in a 24-well-plate for 24 h. Cells were treated with different concentrations of CuCl_2_ (0, 0.2, 0.4 and 0.6mmol/l) for 24 h. Medium was discarded, cells were washed with DPBS (Gibco) and were collected in Qiazol™ solution (Qiagen). RNA isolation was performed by RNeasy Mini Kit (Qiagen) according to manufacturer’s instructions. RNA concentration measurement was performed by spectrophotometry (NanoPhotometer N60 Mobile, IMPLEN GmbH). The cDNA synthesis was performed by QuantiTect Reverse Transcription Kit (Qiagen) according to manufacturer’s instructions using 1 µg RNA. Quantitative polymerase chain reaction was performed using the QuantiNova SYBR Green PCR Kit (Qiagen). For the detection of the reference gene *GAPDH* sense 5′-TCA AGG CTG AGA ACG GGA AG-3′ and antisense 5′-CGC CCC ACT TGA TTT TGG AG-3′ primers were used. *GAPDH* expression itself was not altered under treatment condition (data not shown). For all other genes, QuantiTect Primer Assays were purchased from Qiagen: *DNAJB1* (QT00233345), *FOS* (QT00007070), *GABARAPL1* (QT00096509), *GADD45B* (QT00018480), *HMOX1* (QT00092645), *HSP90AA1* (QT01002603), *JUN* (QT00242956), *JUNB* (QT00201341), *LAMTOR3* (QT00057995), *PLA2G2A* (QT01476097), *PLOD2* (QT00083083), *PRAGC* (QT00086527) and *UBC* (QT00234430).

### GSH/GSSG-Glo™ assay

GSH/GSSG ratio of CuCl_2_-treated HepG2 and HepG2 ATP7B-KO cells was measured by GSH/GSSG-Glo™ Assay (Promega) according to manufactures instructions. 20,000 cells were plated in 100 µl medium in a 96-well-plate for 24 h at 37 °C. After cells reached confluence, incubation with different CuCl_2_ concentrations (0, 0.2 and 0.6mmol/l) was performed for 6 h. Cells were washed with DPBS and were treated with total glutathione lysis reagent or oxidized glutathione lysis reagent (50 µl/well) for 5 min on a plate shaker. Luciferin generation reagent (50 µl/well) was added. The reaction plate was shaken and incubated for 30 min at room temperature. Luciferin detection reagent was added (100 µl/well) and incubated for 15 min at room temperature. Luminescence measurement was performed by multi-mode microplate reader (FLUOstar Omega).

### ROS-Glo™ H_2_O_2_ assay

H_2_O_2_ induction in HepG2 and HepG2 ATP7B-KO cells after CuCl_2_ treatment was performed by ROS-GLO™ H_2_O_2_ Assay (Promega) according to manufacturer’s instructions. 20,000 cells were plated in a 96-well plate for 24 h at 37 °C. CuCl_2_ (0, 0.2, 0.6mmol/l) in 80 µl medium was added. Cells were incubated for 18 h at 37 °C. Then, 25µM H_2_O_2_ substrate solution was added and incubated for 6 h at 37 °C. ROS-Glo detection solution (100 µl/well) was added and incubated for 20 min at room temperature. Medium without cells was measured for the assay background. H_2_O_2_ level is proportional to the luminescent signal which was measured by a multi-mode microplate reader (FLUOstar Omega).

### Protein lysis and LEGENDplex™ assay

Proteins from CuCl_2_-treated HepG2 and HepG2 ATP7B-KO cells were lysed by plating 500,000 cells in 800 µl medium in 12-well plates. After 24 h medium was changed to medium containing CuCl_2_ (0, 0.2, 0.6mmol/l) for 6 h. Cells were washed with DPBS and lysed on ice with 100 µl RIPA-Puffer (ThermoFisher Scientific) supplemented with protease (Roche Diagnostics) and phosphatase (Roche Diagnostics) inhibitors. Cell lysates were centrifuged for 10 min at 14,000 g at 4 °C. Supernatant was collected. Protein concentration was determined by BCA-Assay (ThermoFisher) according to manufacturer’s instruction. Supernatant was taken from HepG2 and HepG2 ATP7B-KO cells treated with CuCl_2_ (0, 0.2, 0.6mmol/l) for 24 h. LEGENDplex™ Assay (Biolegend) was performed using 20 µg protein and 20 µl supernatant in a LEGENDplex™ HU Th Cytokine Panel (BioLegend).

### Plasmid transfection and luciferase reporter assay

The luciferase-based reporter plasmids pNFKB-Luc and pTA-Luc (Takara Bio, San Jose, USA) were applied with Lipofectamine™ 2000 (Thermo Fisher Scientific) according to the manufacturer’s instructions. Promoter activity was determined via the luciferase assay system (Promega) according to the manufacturer’s instructions.

### Multivariance analysis of RNA sequencing data GSE107323

RNA sequencing data GSE107323 [2] were filtered by calculating the mean gene count values for all gene IDs (all conditions). The threshold value was set at > 30. For the principal component analysis (PCA) the variance was filtered by default optimisation settings and a predefined standard deviation of *p* = 0.005 comparing untreated HepG2 and HepG2 ATP7B-KO cells resulted in a false discovery rate (FDR) of q = 0.01788. PCA and a heat map were created with Qlucore Omics Explorer 3.8 (Qlucore, Lund, SE). Vulcano plots were generated by calculating the log_2_(fold change) and -log_10_(p-value) of all genes with a mean gene count value > 30 counts (cell type-specific conditions) using Graphpad Prism (GraphPad Software, Boston, Massachusetts USA). P-values < 0.05 were set as significant. Genes were analysed using function annotation analysis for biological processes by DAVID tool [10, 11].

### Statistical analysis

All statistical analysis was performed using GraphPad Prism version 10.6.1 for Windows. Data are expressed as means with ± standard deviations (SD) from at least three independent experiments. Gene expression was measured one time in biological duplicates and two times in biological triplicates. Experimental group sizes are presented in the figures. Statistical significance was determined using two-way ANOVA tests with Tukey’s multiple comparison test as a post-hoc-test to compare effects of copper concentrations in HepG2 and HepG2 ATP7B-KO cells (* *p* < 0.05; ** *p* < 0.01; *** *p* < 0.001; **** *p* < 0.0001) or to measure differences between HepG2 and HepG2 ATP7B-KO cells after 6–24 h (# *p* < 0.05; ## *p* < 0.01; ### *p* < 0.001; ####; *p* < 0.0001). Differences in gene expression between PHH control and WD cells were tested by an unpaired t-test (× *p* < 0.05; ×× *p* < 0.01; ×××× *p* < 0.0001).

## Results

### Genes related to autophagy, oxidative stress and inflammation are upregulated in HepG2 ATP7B-KO cells: RNA sequencing analysis

**Fig. 1:**
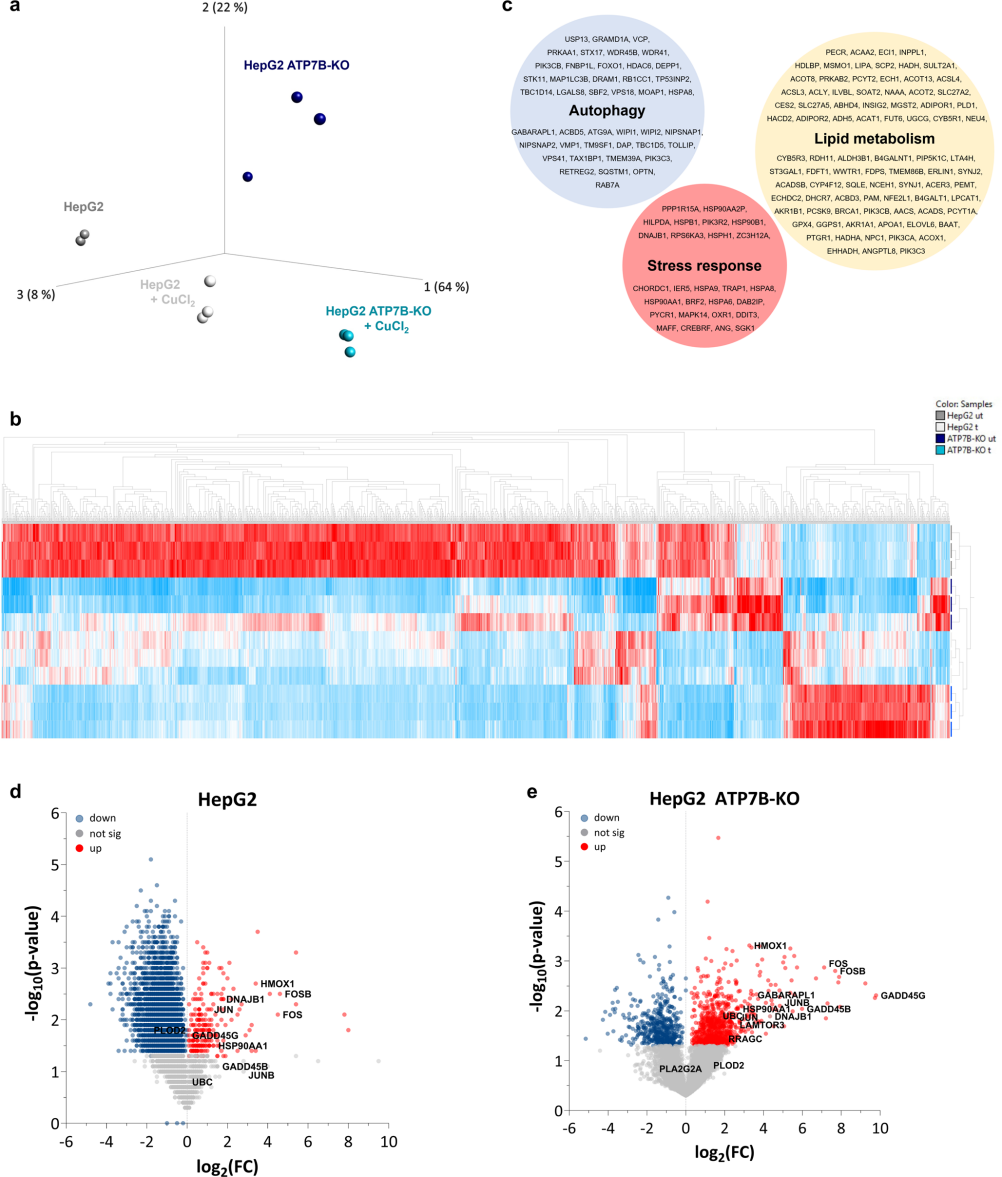
Multivariate analysis of RNA sequencing data. The GSE107323 dataset includes HepG2 and HepG2 ATP7B-KO cells that were treated with 0.5mmol/l CuCl_2_ for 6–24 h. Mean gene counts > 30 were used for analysis. **a** Principal component analysis was performed with the following settings for standard deviation: *p* = 0.005 and q = 0,01788 (FDR). **b** Thus, 2,142 genes were hierarchically clustered via a multivariance analysis visualised in a heatmap. **c** Genes were analysed by functional annotation analysis for biological processes by the DAVID tool and showed high EASE scores in genes involved in autophagy (*p* = 1.1E-7), lipid metabolism (*p* = 3.0E-6) and stress response (*p* = 8,6E-5). Volcano plots were generated to compare the CuCl_2_-dependent gene regulation, indicating **d** 7,722 gene alterations in HepG2 cells and **e** 7,241 gene alterations in HepG2 ATP7B-KO cells. sig, significant; ut, untreated; t, treated

Previously published results indicated that under basal conditions, cellular copper load is approximately fourfold higher in HepG2 ATP7B-KO cells, than in HepG2 cells [2]. While comparable saturated copper levels have been reached after treatment with 0.1mM CuCl_2_, HepG2 wildtype cells more efficiently export copper ions [7]. Furthermore, after copper treatment, intracellular copper levels remain significantly higher in HepG2 ATP7B-KO cells [12]. To investigate the influence of copper and identify different regulatory gene patterns in HepG2 and HepG2 ATP7B-KO cells, RNA sequencing data GSE107323 [2] were reanalysed by calculating the mean gene count values for all gene IDs (including all conditions). By setting a gene count threshold at > 30, a total of 7,661 out of 45,878 genes were selected. To visualise similarities between groups of samples in this data set a principal component analysis (PCA) was performed. The variance was filtered by default optimisation settings and a predefined standard deviation of *p* = 0.005 comparing untreated HepG2 and HepG2 ATP7B-KO resulted in an FDR of q = 0.01788. Thus 2,142 genes remained relevant. The PCA blot including these genes distinguished four different groups, comprising three datasets, with an outlier in the untreated HepG2 ATP7B-KO group (Fig. 1a). Untreated HepG2 cells and copper-treated HepG2 cells were found to be closely clustered. In contrast, untreated HepG2 ATP7B-KO cells were further separated from copper-treated HepG2 ATP7B-KO cells. Furthermore, analysis of these data revealed a closer association between treated HepG2 cells and untreated HepG2 ATP7B-KO cells.

To identify regulatory gene patterns, a hierarchical clustered multivariate analysis of all 2,142 significantly regulated genes was conducted, and a heatmap was created. Untreated HepG2 and HepG2 ATP7B-KO cells showed distinct expression profiles, with most genes upregulated in HepG2 and downregulated in HepG2 ATP7B-KO cells (Fig. 1b). Copper treatment led to similar global downregulation in both cell types, though 409 genes were upregulated in ATP7B-KO cells compared to HepG2 cells. Expression Analysis Systematic Explorer (EASE) analysis linked these genes to stress response (*p* = 6.4E-9), apoptosis (*p* = 1.6E-3), and transcription regulation (*p* = 8.6E-3). Functional enrichment revealed associations with autophagy (*p* = 1.1E-7), lipid metabolism (*p* = 3.0E-6), apoptosis (*p* = 3.0E-5), and stress response (*p* = 8.6E-5) (Fig. 1c).

Volcano plot analysis of the GSE107323 dataset was performed with gene counts > 30. In HepG2 cells 7,722 genes were of interest. Upon copper treatment, 4,784 genes were significantly downregulated, while 248 were upregulated (*p* < 0.05), including genes linked to autophagy (*HSP90AA1*, *PLOD2*), oxidative stress (*HMOX1*, *DNAJB1*, *GADD45B*), and immune responses (*FOS*, *FOSB*, *JUN*) (Fig. 1d). In HepG2 ATP7B-KO cells, 7,241 genes met the threshold, with 481 downregulated and 780 upregulated. Notably, more autophagy-related genes (e.g. *HSP90AA1*, *GABARAPL1*, *UBC*, *PRAGC*, *LAMTOR3*), oxidative stress-related genes (e.g. HMOX1, DNAJB1, GADD45B/G), and immune-related genes (e.g. *FOS*, *FOSB*, *JUN*, *JUNB*) were upregulated compared to HepG2 cells (Fig. 1e).

### The expression of autophagy genes is increased in HepG2 ATP7B-KO and PHH WD cells

**Fig. 2:**
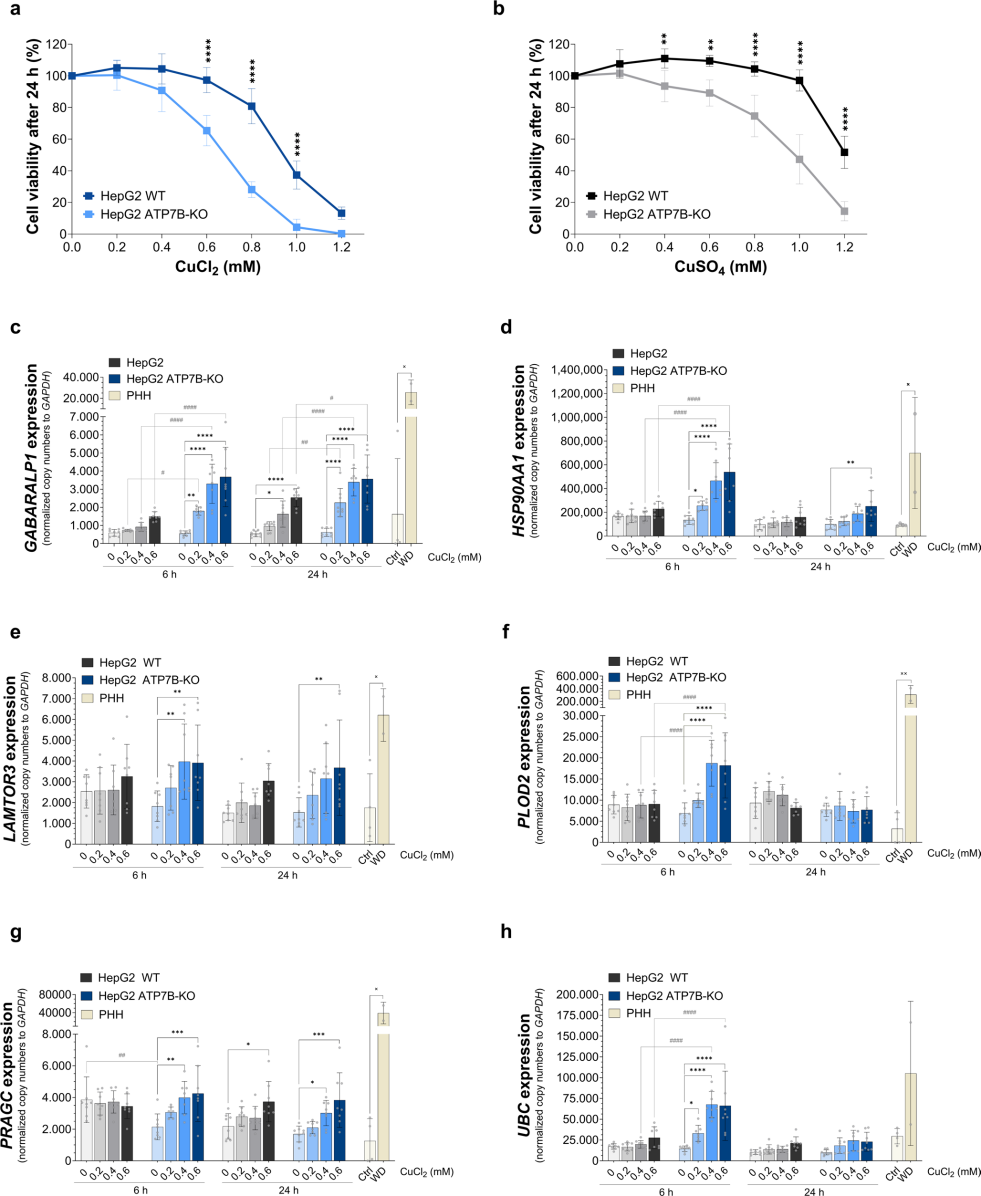
CuCl_2_-dependent gene expression of autophagy-associated genes. HepG2 and HepG2 ATP7B-KO cells were treated with 0.2–1.2.2mmol/l CuCl_2_ or CuSO_4_ for 6–24 h. Cell viability was measured after **a** CuCl_2_ and **b** CuSO_4_ treatment via a CCK-8 assay and normalised to that of the untreated control (mean ± SD; *n* = 4 (CuCl_2_), *n* = 5 (CuSO_4_)). RNA from HepG2 and HepG2 ATP7B-KO cells treated with CuCl_2_ and untreated PHH (Ctrl and WD) was extracted, and the expression of autophagy-related genes (**c**, *GABARALP1*; **d**, *HSP90AA1*; **e**, *LAMTOR3*; **f**, *PLOD2*; **g**, *PRAGC*; **h**, *UBC*) was measured via two-step qRT‒PCR (mean ± SD; *n* = 3 (HepG2 and HepG2 ATP7B-KO), *n* = 4 (PHH Ctrl), *n* = 2 (PHH WD). The data represent copy numbers normalised to 100,000 copies of the reference gene *GAPDH*. The effect of CuCl_2_ concentrations in HepG2 or HepG2 ATP7B-KO cells (* *p* < 0.05; ** *p* < 0.01; *** *p* < 0.001; **** *p* < 0.0001) and differences between HepG2 and HepG2 ATP7B-KO cells after 6–24 h (# *p* < 0.05; ## *p* < 0.01; ### *p* < 0.001; ####; *p* < 0.0001) were measured by two-way ANOVA tests and Tukey’s multiple comparison tests. Differences in gene expression between PHH Ctrl and WD cells were tested by an unpaired t-test (× *p* < 0.05; ×× *p* < 0.01). *Ctrl* control, *PHH* primary human hepatocytes, *WD* Wilson’s disease

To validate the copper-dependent gene regulation in HepG2 and HepG2 ATP7B-KO cells, both were cultured in RPMI medium, contrasting with the GSE107323 dataset, which used DMEM and RPMI medium, respectively. Cytotoxicity assays with CuCl_2_ and CuSO_4_ at varying concentrations for 24 h showed that HepG2 cells had greater viability than HepG2 ATP7B-KO cells. A 50% toxicity rate was reached at 0.94mmol/l in HepG2 and 0.68mmol/l in HepG2 ATP7B-KO cells. Low CuCl_2_ concentrations (0.2-0.2.4mmol/l) did not induce significant differences, while higher concentrations (0.6–1.6.0mmol/l) led to significant toxicity in HepG2 ATP7B-KO cells (Fig. 2a). CuSO_4_-treated HepG2 cells also showed better viability, with 50% toxicity at 1.21mmol/l in HepG2 and 0.98mmol/l in HepG2 ATP7B-KO cells. At 0.2mmol/l CuSO_4_, no significant differences were observed, but higher concentrations (0.4–1.4.2mmol/l) significantly increased toxicity in HepG2 ATP7B-KO cells (Fig. 2b). Since both cell types were more sensitive to CuCl_2_ treatment, it was used for subsequent experiments.

To assess copper-dependent regulation of autophagy-related genes, the expression of six genes (*HSP90AA1*, *GABARALP1*, *UBC*, *PRAGC*, *PLOD2*, *LAMTOR3*) was measured in HepG2 and HepG2 ATP7B-KO cells after CuCl₂ treatment. In HepG2 cells, four genes showed no copper response at 6–24 h, while *GABARALP1* and *PRAGC* were significantly upregulated at 0.6mmol/l after 24 h (*p* < 0.0001 and *p* < 0.05, respectively). In contrast, HepG2 ATP7B-KO cells exhibited significant, dose-dependent upregulation of all six genes after 6 h, with sustained expression of *GABARALP1*, *PRAGC*, and *LAMTOR3* at 24 h. Direct comparison revealed significantly higher expression of *GABARALP1*, *HSP90AA1*, *PLOD2*, and *UBC* in HepG2 ATP7B-KO cells at 6 h, and only *GABARALP1* remained elevated at 24 h. In primary hepatocytes from Wilson disease patients, five of six genes were significantly upregulated compared to controls (*p* < 0.05 or *p* < 0.01), except *UBC*, which showed no significant difference (*p* = 0.1192) (Fig. 2c–h).

### Copper induces oxidative stress and H_2_O_2_ production in HepG2 ATP7B-KO cells

**Fig. 3:**
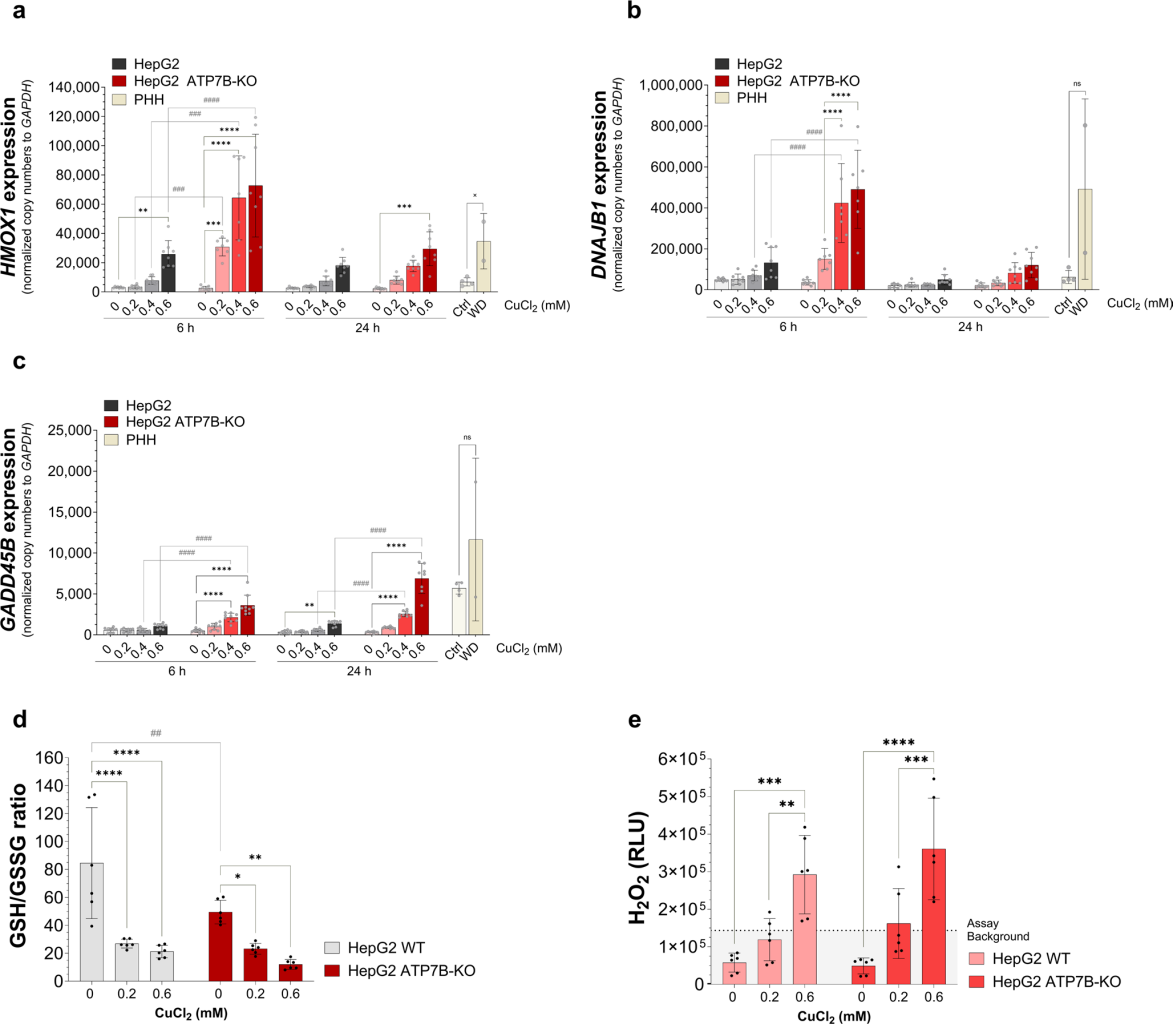
Copper induces oxidative stress and H_2_O_2_ production. HepG2 and HepG2 ATP7B-KO cells were treated with 0.2–0.6mmol/l CuCl_2_ for 6–24 h. RNA from HepG2 and HepG2 ATP7B-KO cells and PHH (Ctrl and WD) was extracted, and the gene expression of oxidated stress-related genes (**a**, *HMOX1*; **b**, *DNAJB1*; **c**, *GADD45B*) was measured via two-step qRT-PCR. The data represent copy numbers normalised to 100,000 copies of the reference gene *GAPDH* (mean ± SD; *n* = 3 (HepG2), *n* = 4 (PHH Ctrl), *n* = 2 (PHH WD)). **d** The GSH/GSSG ratios of HepG2 and HepG2 ATP7B-KO cells were measured via the GSH/GSSG-Glo™ assay (means ± SDs; *n* = 3). **e** H_2_O_2_ levels in HepG2 and HepG2 ATP7B-KO cells were measured by luminescence via the ROS-Glo™ H_2_O_2_ assay (mean ± SD; *n* = 3). The effect of CuCl_2_ concentrations in HepG2 or HepG2 ATP7B-KO cells (* *p* < 0.05; ** *p* < 0.01; *** *p* < 0.001; **** *p* < 0.0001) and differences between HepG2 and HepG2 ATP7B-KO cells after 6–24 h (# *p* < 0.05; ## *p* < 0.01; ### *p* < 0.001; ####; *p* < 0.0001) were measured by two two-way ANOVA tests and a Tukey’s multiple comparison tests. Differences in gene expression between PHH Ctrl and WD cells was tested by an unpaired t-test (× *p* < 0.05; ×× *p* < 0.01). *Ctrl* control, *PHH* primary human hepatocytes, *WD* Wilson’s disease

After 6 h of CuCl₂ treatment, chosen oxidative stress-related genes were significantly upregulated in HepG2 ATP7B-KO cells, while only *HMOX1* showed early induction in HepG2 cells. At 24 h, the upregulation of *HMOX1* and *GADD45B* remained significant in both cell types, but *DNAJB1* regulation declined and was no longer significant in HepG2 ATP7B-KO cells. Direct comparison confirmed significantly higher expression of *HMOX1*, *DNAJB1*, and *GADD45B* in HepG2 ATP7B-KO cells at 6 h, and of *GADD45B* at 24 h (*p* < 0.001 or *p* < 0.0001). In primary hepatocytes, *HMOX1* expression was significantly elevated in the WD group (*p* < 0.05), while *DNAJB1* and *GADD45B* showed a trend toward upregulation that did not reach statistical significance (Fig. 3a–c). These findings indicate that HepG2 ATP7B-KO cells exhibit an earlier and more robust copper-dependent upregulation of oxidative stress-related genes compared to HepG2 cells.

To assess copper-induced oxidative stress, the GSH/GSSG ratio was measured in HepG2 and HepG2 ATP7B-KO cells after 6 h of CuCl₂ treatment (0.2 and 0.6mmol/l). In HepG2 cells, the ratio dropped from 84.58 (untreated) to 26.91 and 21.28 at 0.2 and 0.6mmol/l, respectively (*p* < 0.0001). In HepG2 ATP7B-KO cells, the ratio decreased from 49.48 to 23.28 (0.2mmol/l, *p* < 0.05) and 12.05 (0.6mmol/l, *p* < 0.01). A significant difference between cell lines was observed only under untreated conditions (*p* < 0.01) (Fig. 3d).

Oxidative stress in the context of copper-induced cell toxicity [3, 4] has been linked with the Fenton and Haber-Weiss reactions due to the production of free radicals in conjugation with H_2_O_2_ [13]. Here, a luminescent-based assay was applied to CuCl₂-treated HepG2 and HepG2 ATP7B-KO cells, in which RLU are proportional to H₂O₂ levels (Fig. 3e). Untreated HepG2 cells showed 57,966 RLU, increasing significantly to 119,071 RLU at 0.2mmol/l (*p* < 0.01) and 292,313 RLU at 0.6mmol/l (*p* < 0.001). Untreated HepG2 ATP7B-KO cells reached 49,130 RLU, which increased to 162,087 RLU (*p* < 0.001) and 360,634 RLU (*p* < 0.0001) at respective CuCl₂ concentrations. Background H₂O₂ level was 143,596 RLU.

### Copper induces increased levels of inflammation in HepG2 ATP7B-KO cells

**Fig. 4:**
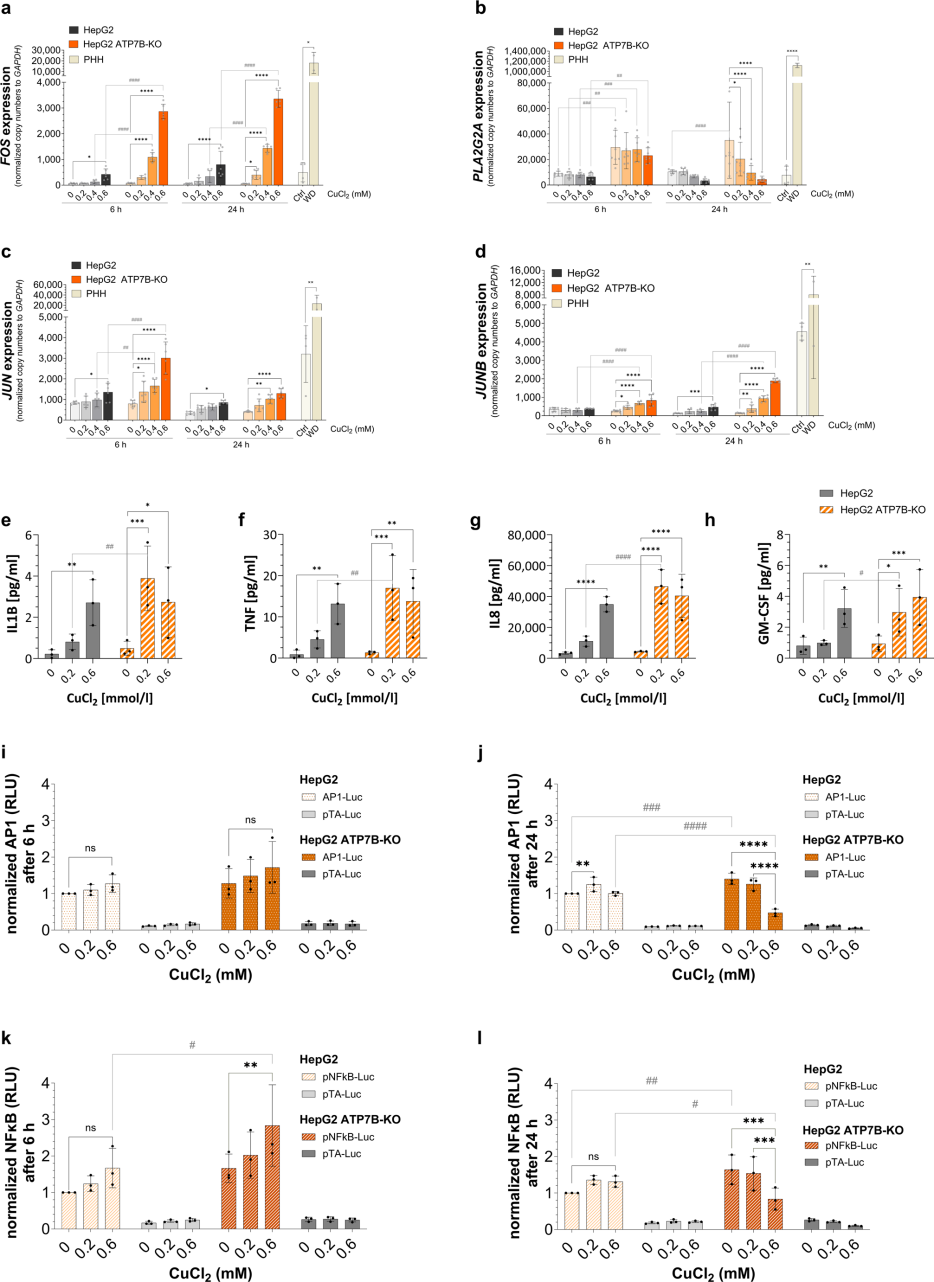
Copper induces NFKB- and AP1-related inflammatory responses. HepG2 and HepG2 ATP7B-KO cells were treated with 0.2-0.2.6mmol/l CuCl_2_ for 6 and 24 h. RNA from HepG2 and HepG2 ATP7B-KO cells and untreated PHH (Ctrl and WD) was extracted, and the gene expression of genes related to NFKB and AP1 (**a**, *FOS*; **b**, *PLA2G2A*; **c**, *JUN;* **d**, *JUNB*) was measured via two-step qRT‒PCR (mean ± SD; *n* = 3 (HepG2), *n* = 4 (PHH Ctrl), *n* = 2 (PHH WD)). Supernatants from HepG2 and HepG2 ATP7B-KO cells treated with CuCl_2_ were collected, and various cytokine levels (**e**, IL1B; **f**, TNF; **g**, IL8; **h**, GM-CSF) were measured via the LEGENDplex™ Assay (mean ± SD, *n* = 3). HepG2 and HepG2 ATP7B-KO cells were transfected with luciferase-based reporter plasmids pAP1-Luc (**i**,** j**), pNFKB-Luc (**k**, **l**) and pTA-Luc as a control and incubated with CuCl_2_. Measurement of NFKB and AP1 promoter activity was performed via a luciferase reporter assay (means ± SDs, *n* = 3). The effect of CuCl_2_ concentrations in HepG2 or HepG2 ATP7B-KO cells (* *p* < 0.05; ** *p* < 0.01; *** *p* < 0.001; **** *p* < 0.0001) and differences between HepG2 and HepG2 ATP7B-KO cells after 6–24 h (#*p* < 0.05; ##*p* < 0.01; ###*p* < 0.001; ####*p* < 0.0001) were measured by two-way ANOVA tests and Tukey’s multiple comparison tests. Differences in gene expression between PHH Ctrl and WD cells were tested by an unpaired t-test (× *p* < 0.05; ×× *p* < 0.01; ×××× *p* < 0.0001). *Ctrl* control, *ns* not significant, *PHH* primary human hepatocytes, *WD* Wilson’s disease

To assess inflammatory responses, the expression of *FOS*, *PLA2G2A*, *JUN*, and *JUNB* was analysed in copper-treated HepG2 and HepG2 ATP7B-KO cells. In HepG2 cells, 0.6mmol/l CuCl₂ significantly upregulated the expression of *FOS* (6 h, *p* < 0.05; 24 h, *p* < 0.0001), *JUN* (6 h and 24 h, *p* < 0.05), and *JUNB* (24 h, *p* < 0.001), while *PLA2G2A* expression was not responsive. In contrast, all three genes (*FOS*, *JUN*, *JUNB*) were significantly upregulated in HepG2 ATP7B-KO cells at both time points, even at 0.2mmol/l. *PLA2G2A* expression showed a distinct pattern, with copper-dependent downregulation in HepG2 ATP7B-KO cells after 24 h (*p* < 0.0001). Overall, expression levels of all four genes were higher in HepG2 ATP7B-KO cells than in HepG2 cells, although at 0.6mmol/l CuCl₂, *PLA2G2A* expression in HepG2 ATP7B-KO cells matched HepG2 levels after 24 h. Similar differences were observed in PHH, where WD PHH showed significantly higher expression of all four genes compared to PHH controls (Fig. 4a–c).

Given that increased expression of immunological genes was detected, various cytokines (LEGENDplex™, BioLegend) were detected via multiplex flow cytometry in copper-treated HepG2 and HepG2 ATP7B-KO cells. Following a 24 h incubation period with CuCl_2_ concentrations ranging from 0.2 to 0.6mmol/l, a significant copper-dependent regulation was detected for four cytokines in the cell culture supernatant of the treated cells. In HepG2 cells, a significant increase in the levels of cytokines (IL1B, TNF, and GM-CSF, *p* < 0.01; IL8, *p* < 0.0001) was measured at a CuCl_2_ dose of 0.6mmol/l in comparison with those in untreated cells. In addition to 0.6mmol/l HepG2 ATP7B-KO cells, 0.2mmol/l HepG2 ATP7B-KO cells presented increased levels of these four markers. A discrepancy was identified between the two cell types for all four cytokines measured at a concentration of 0.2mmol/l. Compared with HepG2 cells, HepG2 ATP7B-KO cells presented significantly elevated levels of all four cytokines (Fig. 4e–h).

To examine AP1 promoter activity following copper-induced upregulation of *FOS*, *FOSB*, *JUN*, and *JUNB*, a luciferase reporter assay was performed in HepG2 and HepG2 ATP7B-KO cells.

After 6 h of treatment with CuCl_2_, no significant dose-dependent changes or differences between cell types were detected (Fig. 4i). At 24 h, HepG2 cells showed increased AP1 promoter activity at 0.2mmol/l (*p* < 0.01), but this effect was absent at 0.6mmol/l (Fig. 4j). In contrast, HepG2 ATP7B-KO cells displayed a dose-dependent reduction in AP1 activity at 24 h, with significant decreases at both 0.2mmol/l (1.11-fold) and 0.6mmol/l (2.9-fold) (*p* < 0.0001). Notably, baseline AP1 activity was 1.4-fold higher in untreated HepG2 ATP7B-KO cells compared to HepG2 cells (*p* < 0.001), a difference not seen at 0.2mmol/l. However, at 0.6mmol/l, AP1 activity was significantly lower in HepG2 ATP7B-KO cells than in HepG2 cells (*p* < 0.0001).

Higher levels of NFKB-associated cytokines (IL1B, IL8, TNF, GM-CSF) were detected in HepG2 ATP7B-KO cells compared to HepG2 cells (Fig. 4e–h). To investigate this further, NFKB promoter activity was measured after 6 h and 24 h of CuCl₂ treatment (0.2 and 0.6mmol/l). After 6 h, HepG2 cells showed no significant increase, while HepG2 ATP7B-KO cells exhibited a significant induction at 0.6mmol/l (*p* < 0.01) (Fig. 4k). At 24 h, no regulation was observed in HepG2 cells, but HepG2 ATP7B-KO cells showed a dose-dependent reduction, with 0.6mmol/l CuCl₂ reducing promoter activity by 50.76%. Baseline NFKB activity was significantly higher in untreated HepG2 ATP7B-KO cells compared to HepG2 cells (*p* < 0.01). After treatment with 0.6mmol/l, HepG2 ATP7B-KO cells showed significantly lower activity than HepG2 cells (*p* < 0.05) (Fig. 4l).

## Discussion

Wilson’s disease is a rare, potentially life-threatening disorder with variable severity, highlighting the need to uncover its molecular basis. While autophagy has been implicated in Wilson’s disease [2], the roles of inflammation and oxidative stress have been debated [13, 14]. To explore gene expression changes, a multivariate reanalysis of the GSE1073233 dataset was performed, revealing major differences between untreated HepG2 and HepG2 ATP7B-KO cells, particularly in autophagy, lipid metabolism, and stress-related pathways. Functional analysis identified copper-induced inflammatory and oxidative stress responses as key mechanisms in the WD cell model.

Contradictory claims exist regarding the presence of free copper ions in copper-overloaded cells. It is generally accepted that excess cytosolic copper is tightly bound by the metallothioneins, preventing the existence of free, unbound copper ions. For free, unbound copper ions to be present in the cell, an increase in the amount of free copper within the cell would be necessary. For the free copper ions to be detectable, the intracellular concentration would need to increase by nine orders of magnitude, which would result in approximately one unbound copper atom per cell [13, 15]. This finding suggests that the formation of free hydroxyl radicals by copper requires the presence of accessible copper ions [13]. Furthermore, even in the event that accessible copper ions result in the formation of free hydroxyl radicals, such radicals have been shown not to cause lipid peroxidation or DNA damage. Instead, free copper ions bind with thiol groups, thereby preventing their release [13, 16].

Following the identification of oxidative stress-related genes (HMOX1, DNAJB1, GADD45B), higher expression levels were observed in HepG2 ATP7B-KO cells compared to HepG2 cells. This prompted investigation into whether HepG2 ATP7B-KO cells exhibit increased oxidative stress, while the role of excess copper in inducing oxidative stress remains debated. Copper enters hepatocytes mainly via DMT1 (Cu²⁺) and CTR1 (Cu⁺) [13, 17]. Intracellularly, copper is tightly bound by proteins like metallothioneins or glutathione (GSH), preventing the presence of free ions, which are cytotoxic. Free copper can undergo redox cycling, leading to the formation of ROS, including hydroxyl radicals (HO·) via Fenton and Harber-Weiss reactions [13, 17]. Disrupted copper homeostasis has been shown to induce oxidative stress in various models [3, 17, 18]. In patients with Wilson’s disease, excessive hepatic copper accumulation similarly leads to oxidative stress [13, 19–22]. Interestingly, cuproptosis is a recently described modality of regulated cell death, in which copper damages key enzymes of the TCA cycle, leading to impaired energy metabolism and subsequent to downstream oxidative stress. In this pathway, copper-induced oxidative stress is thought to arise mainly from interference with mitochondrial enzymes rather than from copper directly generating ROS [23].

The copper concentration used in experiments critically influences cellular responses: low levels (0.1–0.1.2mmol/l) induce physiological/adaptive transcriptional changes, while higher levels (0.4-0.4.6mmol/l) trigger toxic, stress-related responses [24]. In this study, both low (0–0.2mmol/l) and high (0.4–1.2mmol/l) copper concentrations were applied. Oxidative stress, assessed via the GSH/GSSG ratio, increased in both HepG2 and HepG2 ATP7B-KO cells across all copper doses. A significant difference between cell types was only observed in the absence of copper. Given that free cuprous ions catalyse hydroxyl radical formation via the Fenton and Harber–Weiss reactions [13, 17], hydrogen peroxide levels were measured in copper-treated cells. While both cell types showed increased H₂O₂ levels, no intergroup differences emerged. These results suggest that both low and high copper levels induce oxidative stress, with HepG2 ATP7B-KO cells being more sensitive under physiological copper conditions. ROS are able to activate multiple signalling cascades, including MAPK pathways that regulate AP1 and NFKB transcription factors, which govern proliferation, survival, and immune responses [17, 25]. Despite this potential, copper induced only slight AP1 promoter activation in both cell types. Interestingly, a copper-dependent loss of AP1 activity was observed after 24 h in HepG2 ATP7B-KO cells at higher concentrations. Further studies and additional models are needed to clarify whether this reflects increased toxicity or immune activation.

As previously noted, ROS activate the NFKB signalling pathway [17, 25]. Copper’s impact on NFKB regulation is complex and context-dependent, showing both inhibitory and activating effects. It is generally thought to inhibit or have no effect in adaptive immune cells, while activating NFKB in detoxifying organs such as the liver [26]. In vivo, copper exposure increases NFKB activity and proinflammatory cytokines (IL1B, IL8, TNF) in the liver [27]. Similarly, the toxic milk mouse model, which mimics Wilson’s disease, exhibits elevated NFKB levels in early life, followed by a decline [26, 28]. Increased TNF levels, associated with NFKB, have also been observed in WD patients [29] and animal models [14]. In vitro, high copper concentrations (0.4–0.6mM) have been shown to activate NFKB in HepG2 cells [24], and this activation appears crucial for cell survival under copper stress [26]. In the present study, low copper levels did not affect NFKB promoter activity in either HepG2 or HepG2 ATP7B-KO cells, despite increased NFKB-related cytokine levels in the culture medium. This response may be similar to that observed in toxic milk mice [26, 28] or could result from CuCl₂-induced toxicity, as cell viability dropped to 60% at 0.6mmol/l. The underlying mechanisms remain unclear.

In the cell culture model of Wilson’s disease, excessive copper accumulation unleashed a cascade of cellular stress responses, including autophagy, oxidative stress, and inflammation driven in part by the dysregulation of key signalling hubs such as NFKB and AP1. These critical mechanisms behind copper-related cell death underscore the importance of this model for developing molecular targets for future therapeutic strategies aimed at halting WD progression at its core.

## Data Availability

No datasets were generated or analysed during the current study.

## Abbreviations

Ctrl: Control
EASE: Expression analysis systematic explorer
FDR: False discovery rate
GSH: Glutathione
GSSG: Glutathione disulfide
KO: Knockout
MAPK: Mitogen-activated protein kinase
MT: Metallothionein
PCA: Principal component analysis
PHH: Primary human hepatocytes
RLU: Relative light units
ROS: Reactive oxygen species
WD: Wilson’s disease
